# Pneumonia, Meningitis, and Septicemia in Adults and Older Children in Rural Gambia: 8 Years of Population-Based Surveillance

**DOI:** 10.1093/cid/ciac603

**Published:** 2022-07-29

**Authors:** Edward W Green, Malick Ndiaye, Ilias M Hossain, Yekini A Olatunji, Shah M Sahito, Rasheed Salaudeen, Henry Badji, Ahmed Manjang, Lamin Ceesay, Philip C Hill, Brian Greenwood, Grant A Mackenzie

**Affiliations:** Medical Research Council Unit The Gambia, London School of Hygiene and Tropical Medicine, Fajara, The Gambia; Liverpool School of Tropical Medicine, Liverpool, United Kingdom; Medical Research Council Unit The Gambia, London School of Hygiene and Tropical Medicine, Fajara, The Gambia; Medical Research Council Unit The Gambia, London School of Hygiene and Tropical Medicine, Fajara, The Gambia; Medical Research Council Unit The Gambia, London School of Hygiene and Tropical Medicine, Fajara, The Gambia; Medical Research Council Unit The Gambia, London School of Hygiene and Tropical Medicine, Fajara, The Gambia; Medical Research Council Unit The Gambia, London School of Hygiene and Tropical Medicine, Fajara, The Gambia; Medical Research Council Unit The Gambia, London School of Hygiene and Tropical Medicine, Fajara, The Gambia; Medical Research Council Unit The Gambia, London School of Hygiene and Tropical Medicine, Fajara, The Gambia; Ministry of Health and Social Welfare, Banjul, The Gambia; Centre for International Health, University of Otago, Dunedin, New Zealand; Faculty of Infectious and Tropical Diseases, London School of Hygiene and Tropical Medicine, London, United Kingdom; Medical Research Council Unit The Gambia, London School of Hygiene and Tropical Medicine, Fajara, The Gambia; Faculty of Infectious and Tropical Diseases, London School of Hygiene and Tropical Medicine, London, United Kingdom; Murdoch Children’s Research Institute, Melbourne, Australia

**Keywords:** pneumonia, sepsis, meningitis, epidemiology, Africa

## Abstract

**Background:**

Representative data describing serious infections in children aged ≥5 years and adults in Africa are limited.

**Methods:**

We conducted population-based surveillance for pneumonia, meningitis, and septicemia in a demographic surveillance area in The Gambia between 12 May 2008 and 31 December 2015. We used standardized criteria to identify, diagnose, and investigate patients aged ≥5 years using conventional microbiology and radiology.

**Results:**

We enrolled 1638 of 1657 eligible patients and investigated 1618. Suspected pneumonia, septicemia, or meningitis was diagnosed in 1392, 135, and 111 patients, respectively. Bacterial pathogens from sterile sites were isolated from 105 (7.5%) patients with suspected pneumonia, 11 (8.1%) with suspected septicemia, and 28 (25.2%) with suspected meningitis. *Streptococcus pneumoniae* (n = 84), *Neisseria meningitidis* (n = 16), and *Staphylococcus aureus* (n = 15) were the most common pathogens. Twenty-eight (1.7%) patients died in hospital and 40 (4.1%) died during the 4 months after discharge. Thirty postdischarge deaths occurred in patients aged ≥10 years with suspected pneumonia. The minimum annual incidence was 133 cases per 100 000 person-years for suspected pneumonia, 13 for meningitis, 11 for septicemia, 14 for culture-positive disease, and 46 for radiological pneumonia. At least 2.7% of all deaths in the surveillance area were due to suspected pneumonia, meningitis, or septicemia.

**Conclusions:**

Pneumonia, meningitis, and septicemia in children aged ≥5 years and adults in The Gambia are responsible for significant morbidity and mortality. Many deaths occur after hospital discharge and most cases are culture negative. Improvements in prevention, diagnosis, inpatient, and follow-up management are urgently needed.

## Teaser

Disease incidence and outcome calculations need prospective population-based data and postdischarge follow-up. We use 8 years of surveillance to highlight the etiology, incidence, outcome, and high burden of pneumonia, sepsis, and meningitis in older children and adults. in rural Gambia.

Meningitis, septicemia, and pneumonia are leading killers worldwide; 48.9 million cases of sepsis occur each year [1]. Data from low- and middle-income countries on the epidemiology of these infections usually focus on children <5 years of age [2–11], and there is a paucity of robust data for older children and adults [12–19]. Many studies in sub-Saharan Africa are hospital based, thus excluding most pneumonia cases and without denominator data, and therefore are unable to calculate the population incidence of disease; many studies are human immunodeficiency virus (HIV) focused, limiting their generalizability. None have reported outcomes postdischarge. To determine the magnitude of the problem and etiology of pneumonia, meningitis, and septicemia in the population, among older children and adults in sub-Saharan Africa, prospective, population-based studies are needed. Therefore, we conducted 8 years of standardized, population-based surveillance for meningitis, septicemia, and pneumonia in all age groups in rural Gambia. We report here the etiology, minimum incidence, and outcome of pneumonia, meningitis, and septicemia in patients aged ≥5 years in a typical African setting.

## METHODS

### Study Setting

**Figure 1.**
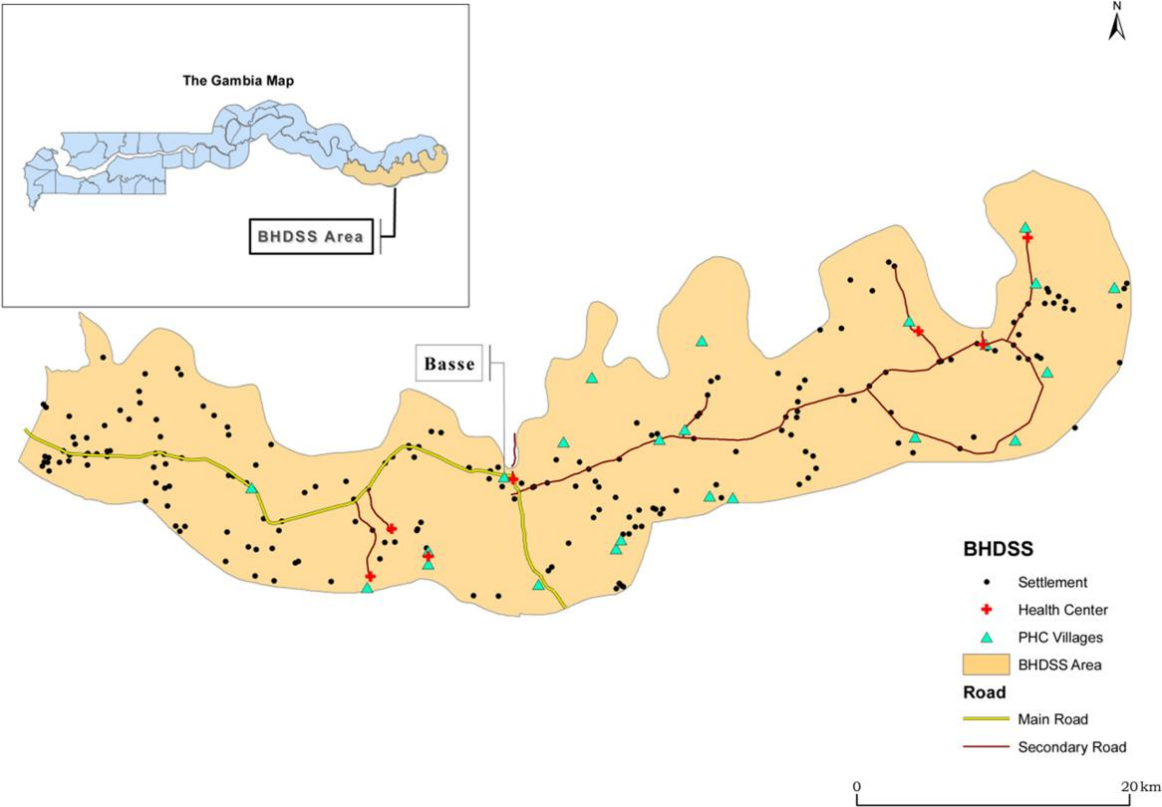
Area covered by the Basse Health and Demographic Surveillance System with inset showing map of Gambia. Abbreviations: BHDSS, Basse Health and Demographic Surveillance System; PHC, primary health centre.

The Gambia is a country of 1.8 million people in West Africa. In 2019, the World Bank estimated the gross domestic product per capita as US$2321. The climate is typical of the sub-Sahel with a short rainy season and a longer dry season. The Upper River Region (URR) is the most eastern and remote part of the country (Figure 1). The major health center in URR is Basse Health Centre, which admits patients directly and takes referrals from smaller facilities in the region.

### Study Population

The Basse Health and Demographic Surveillance System (BHDSS) operates in the half of URR that lies to the south of the Gambia River (population 171 070 in 2012). The population is largely rural, and the major occupation is agriculture. This study included all residents of the BHDSS, the population of which is enumerated every 4 months with records of births, deaths, and migrations. The prevalence of HIV infection among women in antenatal care in 2012 was 1.2% [20]. This report is restricted to patients aged ≥5 years who were confirmed resident in the BHDSS.

Pneumococcal conjugate vaccine (PCV), which was introduced for infants in the URR as 7-valent PCV in August 2009 and as 13-valent PCV in May 2011. A previous analysis indicates that the incidence of invasive pneumococcal disease during this study period showed a possible trend toward herd protection for older patients, but data were insufficient to be certain [21].

### Case Definitions and Study Procedures

The methods used in this study have been described previously [21, 22]. In brief, we conducted 24 hours a day surveillance for pneumonia meningitis and septicemia from 12 May 2008 to 31 December 2015, screening all individuals who presented to Basse Health Centre or to one of the peripheral healthcare facilities in the region, whether or not they were admitted. Surveillance nurses applied standard criteria to every patient to identify cases for referral to a clinician at Basse Health Centre (Table 1 and Supplementary Appendix 1.1). Clinicians applied standard criteria to generate a surveillance diagnosis of suspected meningitis, septicemia, pneumonia, or another diagnosis (Table 1 and Supplementary Appendix 1.2). The surveillance diagnosis led to standardized investigation with blood culture, lumbar puncture, and chest radiography (Table 1 and Supplementary Appendix 1.3). According to clinical and radiological features, aspiration of pleural fluid or lung aspiration was performed for patients with pleural effusion or dense peripheral pneumonic consolidation. Weight was measured using a digital scale (HD-314, Tanita, Arlington Heights, Illinois) and height using a ShorrBoard (Weigh and Measure, LLC, Olney, Maryland). In 2010, samples were not collected between 5 October and 3 November, and radiographs were not performed between 5 and 12 October, when the Gambia River flooded the Medical Research Council (MRC) Unit Field Station in Basse. Patients were admitted or treated as outpatients according to national guidelines. The outcome of each patient’s admission was recorded. Postdischarge mortality was monitored through visits every 4 months to each household in the BHDSS.

**Table 1.** Abbreviated Nurse Screening and Physician Diagnostic Criteria and Standard Investigations According to Disease

Study stage	Criteria used for study enrolment or standardised investigation according to suspected disease
Screening	One or more of the following has been present for ≤14 days: history of cough and difficulty breathing; history of cough and pleuritic chest pain; history of cough and supraclavicular/sternal recession or nasal flaring; history of productive cough and fever; history of rigors; history of seizure; impaired consciousness; altered mental state; axillary temperature of at least 38°C or <36°C in a patient admitted or being admitted; photophobia; neck stiffness; local musculoskeletal swelling or tenderness; irrespective of residential location, any patient with suspected meningitis
Diagnosis	All 3 suspected illnesses defined as physician diagnoses and also to be considered if: • For meningitis: ≥2 of the following are present: axillary temperature ≥37.5°C; meningism (neck stiffness and/or photophobia); altered mental state (Glasgow Coma Scale <14) • For suspected pneumonia: an illness of ≤14 days’ duration where ≥2 of the following are present: cough; hemoptysis; pleuritic chest pain; breathlessness; axillary temperature of ≥38.0°C • For suspected septicemia: at least 1 of: clinician diagnosis of focal sepsis (including but not limited to: septic arthritis, osteomyelitis, endocarditis, peritonitis, liver abscess, soft tissue abscess or cellulitis) or of generalized septicemia; axillary temperature <36.0°C or ≥38°C in a patient admitted or being admitted; history of rigors
Investigation	All patients are to have blood culture and a rapid diagnostic test for malaria. • Patients with suspected meningitis are to have lumbar puncture. • Patients with suspected pneumonia are to have chest radiograph. • Chest radiograph should also be considered in patients with meningitis or septicemia if the clinician’s impression is of coexisting pneumonia or if it is judged that a chest radiograph will assist in management. • Lung aspirate should be considered for a patient if peripheral consolidation has been demonstrated, preferably by radiograph. • Other investigations including pleural tap and joint aspirate may be considered according to the clinical indication.

### Microbiology

Blood, lung aspirate, cerebrospinal fluid (CSF), pleural fluid, and other microbiological samples were processed at the MRC Basse Field Station using standard methods [23]. Aerobic and anaerobic blood cultures were taken for each adult patient, with 5 mL of blood added to each bottle. Children had 2–3 mL added to a single pediatric blood culture bottle. Bottles were weighed before and after blood was added to ensure adequate filling, with weights reported to clinicians. Biochemical tests, including the Analytical Profile Index (bioMérieux, UK), and serological tests were used to confirm suspected pathogens. Latex agglutination tests were used on all CSF samples to identify *Streptococcus pneumoniae*, *Neisseria meningitidis*, and group B streptococci. Results were aggregated with culture-positive cases. Pneumococcal isolates were serotyped at the MRC Fajara laboratory using a latex agglutination assay employing factor and group-specific antisera (Statens Serum Institut, Copenhagen, Denmark) and polymerase chain reaction [24]. The laboratories in Basse and Fajara submitted to external quality assurance throughout the study (UK National External Quality Assessment Service, World Health Organization [WHO] reference laboratory in Denmark, and the Royal Australasian College of Pathologists).

### Radiology

Chest radiographs were performed using consistent methods producing digital images in accordance with WHO recommendations [25]. Two readings of each radiograph were undertaken by 3 independent readers and discordant results were resolved by a pediatric radiologist with experience in reading adult films. All readers were trained and used the WHO panel of standardized radiographs for calibration to the WHO standard for radiological pneumonia, achieving κ agreement scores of ≥0.8 before reading study radiographs.

### Ethics Approval

The study was approved by The Gambia Government/MRC Institutional Ethics Committee (number 1087) and the ethics committee of the London School of Hygiene and Tropical Medicine. Participants, or their parents or guardians, gave written informed consent.

### Statistical Analysis

Categorical data were compared using the χ^2^ or Fisher exact test. Continuous data were compared using *t* tests with appropriate distributional assumptions. We calculated age-specific annual minimum incidence of disease by dividing the number of cases by the midpoint population estimates from the BHDSS and multiplying by 100 000. We did not adjust our figures for the sensitivity of diagnostic tests, study enrollment rates, or health-seeking behavior. Age groups were prespecified as 5–9 years, 10–14 years, 15–54 years, and ≥55 years. Incidence and 95% confidence intervals (CIs) were calculated assuming a Poisson distribution. Statistical significance was set at a *P* value < .05.

## RESULTS

**Figure 2.**
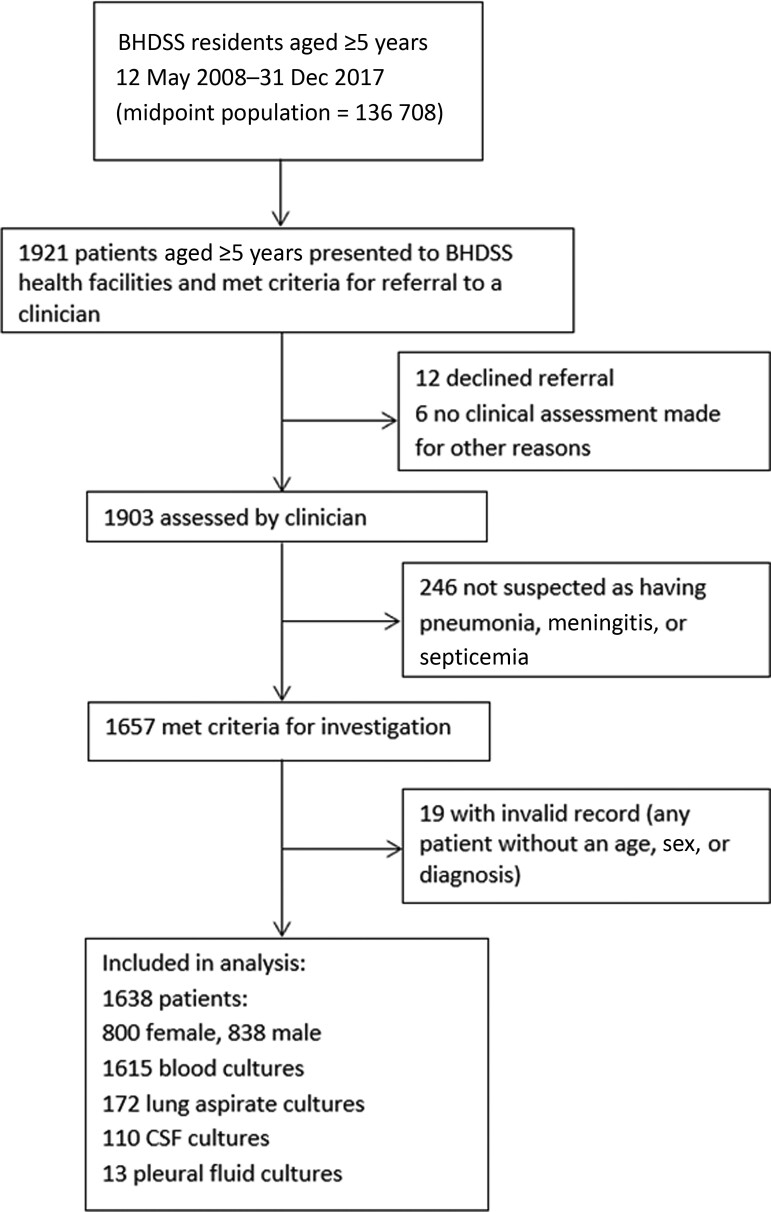
Study profile. Abbreviations: BHDSS, Basse Health and Demographic Surveillance System; CSF, cerebrospinal fluid.

**Figure 3.**
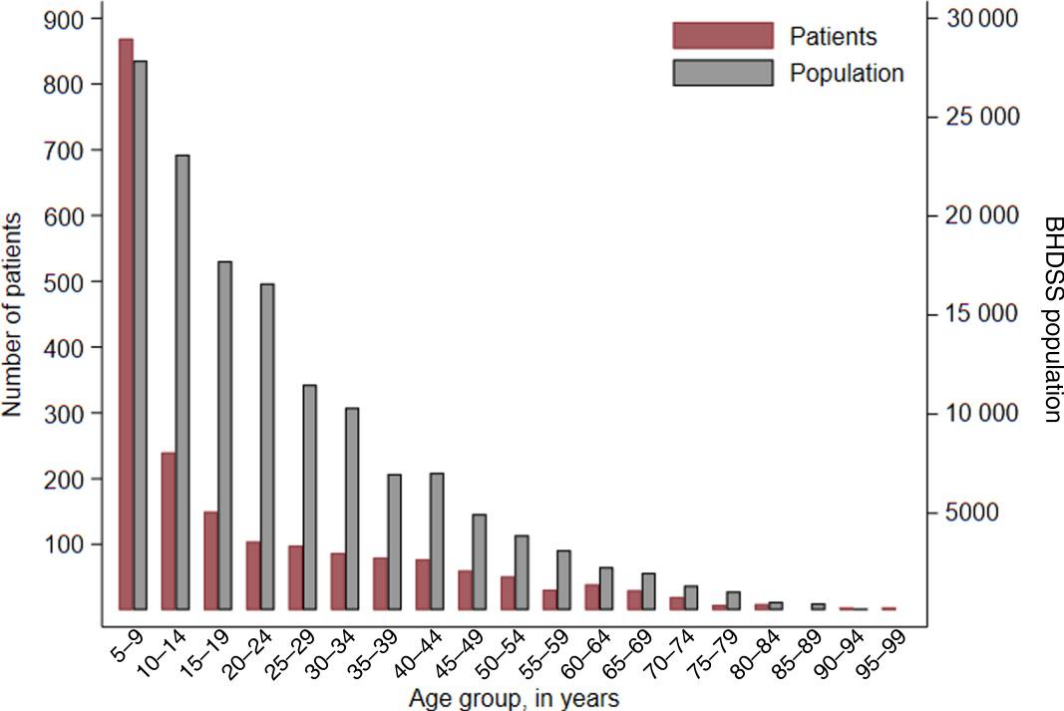
Age distribution of patients ≥5 years of age with suspected pneumonia, septicemia, or meningitis from 12 May 2008 until 31 December 2015 (left y-axis) and the age distribution of the population of the Basse Health and Demographic Surveillance System (BHDSS) in 2011 (right y-axis).

A total of 1921 patients were screened for inclusion and 1657 met inclusion criteria; 19 had invalid records, so 1638 were included in the analysis (Figure 2). The median age of patients was 10.8 years (interquartile range [IQR], 6.8–29 years) with a left-skewed age distribution similar to that of the background population; this was particularly evident in the age group 5–9 years, in which a disproportionate number of cases occurred (Figure 3).

The number of cases of suspected pneumonia (n = 1392) was much greater than those of suspected meningitis (n = 135) or septicemia (n = 111) (Tables 2 and 3). The substantial number of suspected meningitis cases was associated with an epidemic of meningococcal meningitis in the region in 2012 [26]. The nutritional status of patients was poor, with a median body mass index of 14 kg/m^2^ (Table 3). Approximately half of the patients with suspected pneumonia were admitted to hospital.

**Table 2.** Demographic Features of 1638 Patients by Clinical Diagnosis^a^

Characteristic	Suspected Pneumonia	Suspected Meningitis^b^	Suspected Septicemia
	(n = 1392)	(n = 135)	(n = 111)
Age group, y			
ȃ5–9 (n = 762)	617 (44)	97 (72)	48 (43)
ȃ10–14 (n = 204)	170 (12)	17 (13)	17 (15)
ȃ15–54 (n = 562)	500 (36)	18 (13)	44 (40)
ȃ≥55 (n = 110)	105 (8)	3 (2)	2 (2)
Male sex	705 (51)	75 (56)	58 (52)
Town resident^c^	417 (30)	43 (32)	38 (34)
Village resident	975 (70)	92 (68)	73 (66)
No. of people in household, median (IQR)	26 (14–43)	28 (12–41)	23 (12–37)
Annual household income, USD, median (IQR)	300 (75–975)	262 (75–900)	225 (75–900)
Ethnic origin			
ȃSerahule	482 (35)	38 (28)	38 (34)
ȃMandinka	432 (31)	44 (33)	27 (24)
ȃFula	398 (29)	42 (31)	38 (34)
ȃWolof	13 (1)	0 (0)	2 (2)
ȃOther	29 (2)	4 (3)	5 (5)
ȃUnknown	38 (3)	7 (5)	1 (1)

Data are presented as No. (%) unless otherwise indicated.

Abbreviations: IQR, interquartile range; USD, United States dollars.

Diagnostic categories are mutually exclusive; epidemiological variables grouped together are mutually exclusive. Percentages are rounded to the nearest integer.

Twenty-four patients with meningitis were judged by the treating physician to have meningitis and pneumonia.

A town resident is defined as any patient living in a settlement of >5000 people, of which there were 7 in the study area.

**Table 3.** Clinical Features of 1638 Patients by Clinical Diagnosis^a^

Characteristic	Suspected Pneumonia	Suspected Meningitis^b^	Suspected Septicemia
	(n = 1392)	(n = 135)	(n = 111)
Dyspnea	1092 (78)	24 (18)	24 (21)
Cough	1373 (99)	53 (40)	61 (55)
Fever	1303 (94)	129 (96)	107 (96)
Headache	779 (57)	90 (71)	72 (69)
Temperature, °C, median (IQR)	37.4 (36.7–38.6)	37.8 (36.8–38.9)	38.1 (36.9–39.1)
Pulse rate, beats/min, median (IQR)	118 (101–133)	120 (102–140)	118 (98–136)
Respiratory rate, breaths/min, median (IQR)	38 (30–49)	32 (26–42)	30 (26–39)
Oxygen saturation, %, median (IQR)	97 (95–99)	98 (97–99)	98 (97–100)
Meningism	31 (2)	67 (52)	4 (4)
Abnormal conscious state^c^	13 (1)	65 (50)	6 (5)
BMI, kg/m^2^, median (IQR)	14 (13–19)	13 (12–15)	14 (13–19)
Admitted^d^	659 (47.0)	133 (99)	68 (61)

Data are presented as No. (%) unless otherwise indicated.

Abbreviations: BMI, body mass index; IQR, interquartile range.

Diagnostic categories are mutually exclusive. Clinical features are not mutually exclusive; percentages are for those who have data to report. Variables with missing data are as follows: dyspnea, n = 1; cough, n = 1; fever, n = 1; headache, n = 40; temperature, n = 2; pulse rate, n = 1; respiratory rate, n = 0; oxygen saturation, n = 3; meningism, n = 43; abnormal conscious state, n = 9; BMI, n = 107.

Twenty-four patients with meningitis were judged by the treating physician to have meningitis and pneumonia.

Abnormal consciousness assessed using an “AVPU” score: Alert, responding to Voice, responding to Pain, Unresponsive. Any state not “A” is deemed abnormal.

Admission status was undetermined in 1 patient.

**Figure 4.**
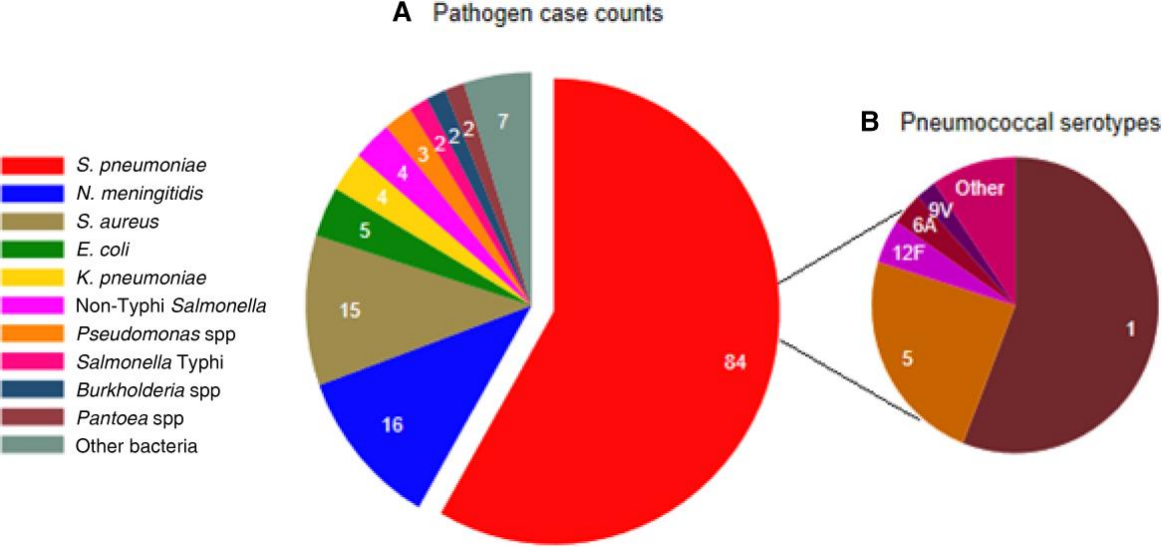
Distribution of bacterial pathogens (*A*) and pneumococcal serotypes (*B*) isolated from patients aged ≥5 years with suspected meningitis, septicemia, or pneumonia in the Basse Health and Demographic Surveillance System between 12 May 2008 and 31 December 2015. *A*, Slices of the pie represent and are labeled with counts of patients from whom different pathogens were isolated, with the pneumococcal sector separated. *B*, Slices of the pie represent counts of patients labeled with the pneumococcal serotypes isolated. Abbreviations: *E. coli*, *Escherichia coli*; *K. pneumoniae*, *Klebsiella pneumoniae*; *N. meningitidis*, *Neisseria meningitidis*; *S. aureus*, *Staphylococcus aureus*; *S. pneumoniae*, *Streptococcus pneumoniae*.

Sterile site specimens were collected from 1618 of 1638 patients (Table 4). Bacterial pathogens were isolated from 144 patients (8.9%). Blood culture positivity was 7.3% (118/1615). CSF was culture positive in 12 of 110 (10.9%) samples tested, pleural fluid in 2 of 13 (15.4%), and lung aspirates in 36 of 172 (20.9%). *Streptococcus pneumoniae* was the predominant pathogen, isolated from 84 patients (5.2%), with *N. meningitidis* (n = 16) and *Staphylococcus aureus* (n = 15) being the next most prevalent pathogens (Table 4, Figure 4). *Streptococcus pneumoniae* was isolated from 5.8% (n = 80) of patients with pneumonia, 2.3% (n = 3) with meningitis, 0.9% (n = 1) with suspected septicemia, and 13.7% (n = 66) with radiological pneumonia with consolidation. Pneumococcal serotypes 1 (46/84 [55%]) and 5 (19/84 [23%]) predominated (Figure 4).

**Table 4.** Bacterial Culture–Positive Disease in 1618 Patients With at Least 1 Sterile Site Specimen Taken^a^

Organism	Suspected Pneumonia	Suspected Meningitis^b^	Suspected Septicemia	All Syndromes
	(n = 1375)	(n = 133)	(n = 110)	(n = 1618)
*Streptococcus pneumoniae*	80 (5.8)	3 (2.3)	1 (0.9)	84 (5.2)
*Staphylococcus aureus*	7 (0.5)	3 (2.3)	5 (4.5)	15 (0.9)
*Salmonella* spp	6 (0.4)	0 (0.0)	2 (1.8)	8 (0.4)
*Escherichia coli*	3 (0.2)	0 (0.0)	1 (0.9)	4 (0.2)
*Neisseria meningitidis* ^c^	0 (0.0)	16 (12.0)	0 (0.0)	16 (1.0)
Other gram negative^d^	9 (0.7)	6 (4.6)	2 (1.8)	18 (1.1)
Total positive	105 (7.6)	28 (21.1)	11 (10.0)	144 (8.9)

Data are presented as No. (%).

Specimens were not collected from 17 of 1392 pneumonia patients, 2 of 135 meningitis patients, and 1 of 111 septicemia patients.

Cerebrospinal fluid from all patients had antigen testing performed for *N. meningitidis*, *S. pneumoniae*, and group B streptococci. Positive antigen tests in patients otherwise culture negative are included in this column.

All *N. meningitidis* were serotype W135.

Pneumonia: *Pseudomonas* spp (2), *Pantoea* spp (1), *Moraxella* spp (1), *Klebsiella* spp (1), *Burkholderia* spp (2), unidentified coliform (2). Meningitis: *Serratia* spp (1), *Pseudomonas* spp (1), *Klebsiella* spp (1), *Haemophilus influenzae* (1), other *Haemophilus* spp (1), *Aeromonas* (1). Septicemia: *Klebsiella* spp (2), *Aeromonas* (1).

Table 5 shows that overall inpatient case fatality was 1.7% (28/1638) and was greatest for patients with suspected meningitis (11.1%), although the absolute numbers of inpatient deaths were similar for suspected meningitis (n = 15) and pneumonia (n = 13). Overall, combining inpatient deaths from all syndromes, the age group with the highest inpatient mortality was ≥55 years (5/110 [4.6%]). Inpatient death was associated with confirmed invasive bacterial disease; 9 of 144 (6.3%) patients with positive cultures died, whereas 19 of 1474 (1.3%) with negative cultures died. Ten of 13 deaths in suspected pneumonia were associated with radiological pneumonia, and 10 of 469 (2.1%) with consolidation died compared to 2 of 827 (0.2%) without consolidation (*P* = .001).

**Table 5.** Inpatient and 4-Month Mortality by Clinical or Radiological Diagnosis^a^

Characteristic	Suspected Pneumonia		Suspected Meningitis		Suspected Septicemia		Radiological Pneumonia	
	Inpatient Mortality	4-Month Mortality	Inpatient Mortality	4-Month Mortality	Inpatient Mortality	4-Month Mortality	Inpatient Mortality	4-Month Mortality
Age group								
ȃAll ages	13/1392 (0.9)	45/1392 (3.2)	15/135 (11.1)	22/135 (16.3)	0/111 (0.0)	1/111 (0.9)	11/482 (0.9)	27/482 (5.6)
ȃ5–9 y	1/617 (0.2)	3/617 (0.5)	14/97 (14)	18/97 (19)	0/48 (0)	1/48 (2)	2/200 (1.0)^b^	3/200 (1.5)
ȃ10–14 y	0/170 (0.0)	5/170 (2.9)	0/17 (0)	1/17 (6)	0/17 (0)	0/17 (0)	0/63 (0)	1/63 (2)
ȃ15–54 y	7/500 (1.4)	21/500 (4.2)	1/18 (6)	2/18 (11)	0/44 (0)	0/44 (0)	6/164 (3.7)	13/164 (7.9)
ȃ≥55 y	5/105 (4.8)	16/105 (15.2)	0/3 (0)	1/3 (33)	0/2 (0)	0/2 (0)	3/55 (5)	10/55 (18)
Admitted	13/659 (2)	29/659 (4.4)	15/133 (11.3)	22/133 (16.5)	0/68 (0)	1/68 (2)	11/380 (2.9)	21/380 (5.5)
Not admitted	NA	16/733 (2.2)	NA	0/2 (0)^c^	NA	0/43 (0)	NA	6/102 (5.9)
Culture result^d^								
ȃ*Streptococcus pneumoniae*	4/80 (5)	6/80 (8)	0/3 (0)	0/3 (0)	0/1 (0)	0/1 (0)	4/66 (6)	6/66 (9)
ȃ*Staphylococcus aureus*	0/7 (0)	1/7 (14)	0/3 (0)	0/3 (0)	0/5 (0)	1/5 (20)	0/4 (0)	1/4 (25)
ȃ*Neisseria meningitidis*	0/0 (0)^e^	0/0 (0)^e^	3/16 (19)	3/16 (19)	0/0 (0)^e^	0/0 (0)^e^	0/5 (0)	0/5 (0)
ȃOther gram negative	0/12 (0)	1/12 (8)	2/6 (33)	2/6 (33)	0/4 (0)	0/4 (0)	0/4 (0)	0/4 (0)
ȃAny pathogen	4/105 (4)	8/105 (8)	5/28 (18)	5/28 (18)	0/11 (0)	1/11 (9)	4/75 (5)	7/75 (9)
ȃCulture negative	9/1270 (1)	37/1270 (3)	10/105 (10)	17/105 (16)	0/99 (0)	0/99 (0)	7/407 (2)	20/407 (5)
Radiology								
ȃRadiological pneumonia with consolidation	10/469 (2.1)	26/469 (5.5)	1/9 (11)	1/9 (11)	0/4 (0)	0/4 (0)	NA	NA
ȃNormal CXR	2/827 (0.2)	15/827 (1.8)	4/34 (12)	7/34 (21)	0/25 (0)	0/25 (0)	NA	NA

Data are presented as no./No. (%).

Abbreviations: CXR, chest radiograph; NA, not applicable.

Inpatient mortality is calculated as a percentage of the whole cohort, not as a percentage of those admitted, and 4-month mortality is also calculated as a percentage of the whole cohort and therefore includes inpatient and outpatient deaths.

One of the 2 patients in this category had coexisting suspected meningitis.

Two patients with suspected meningitis refused admission and survived to 4 months.

Numbers are of patients who had at least 1 sterile site specimen culture.

No cultures were positive for *N meningitidis* in patients with suspected pneumonia or suspected septicaemia.

We found 40 postdischarge deaths compared to 28 inpatient deaths (Table 5). Mortality by 4 months post discharge was 4.1% (68/1638), 2.4 (95%CI, 1.6–3.8) times that of inpatient mortality (Table 5). Four-month mortality was greatest for those with suspected meningitis (22/135 [16.3%]), although the absolute number of deaths associated with suspected pneumonia was greater (n = 45). The increased risk of 4-month mortality compared to inpatient mortality was greatest for patients with radiological pneumonia (5.6% [27/482] vs 0.9% [11/482]). Subgroups with the greatest 4-month mortality were children aged 5–9 years with suspected meningitis (18/97 [19%]) and those aged ≥55 years with radiological pneumonia (10/55 [18%]) or suspected pneumonia (16/105 [15.2%]).

The minimum estimated annual incidence of suspected pneumonia was 133 (95% CI, 126–140) cases per 100 000 person-years (PY) (Table 6). Four hundred eighty-two of the pneumonia cases had radiological consolidation consistent with the WHO standard, giving a minimum annual incidence for radiological pneumonia of 46 (95%CI, 42–50) per 100 000 PY. The highest incidence of suspected pneumonia and radiological pneumonia with consolidation was among children aged 5–9 years: 310 (95%CI, 286–336) and 90 (95%CI, 78–104) per 100 000 PY, respectively. In contrast, the incidence of mortality associated with suspected pneumonia was greatest among those aged 15–54 years (4 [95%CI, 2–5] per 100 000 PY) and ≥55 years (19 [95%CI, 10–31] per 100 000 PY). The minimum incidence of suspected meningitis and septicemia was greatest in children aged 5–9 years: 49 (95% CI, 40–60) and 24 (95% CI, 18–32) cases per 100 000 PY, respectively (Table 6). The incidence of invasive pneumococcal disease (8 [95%CI, 6–10] cases per 100 000 PY) was greater than for any other pathogen.

**Table 6.** Minimum Annual Incidence of Suspected Meningitis, Septicemia, and Pneumonia, Associated 4-Month Mortality, and Invasive Bacterial Disease in the Basse Health and Demographic Surveillance System^a,b^

Age Group	No. of Cases	Incidence per 100 000 PY (95% CI)	No. of Deaths	Incidence per 100 000 PY (95% CI)
All ages				
ȃSuspected pneumonia	1392	133 (126–140)	45	4 (3–6)
ȃSuspected meningitis	135	13 (11–15)	22	2 (1–3)
ȃSuspected septicemia	111	11 (9–13)	1	<1 (<1–1)
ȃRadiological pneumonia with consolidation	482	46 (42–50)	27	3 (2–4)
Age 5–9 y				
ȃSuspected pneumonia	617	310 (286–336)	3	1 (<1–4)
ȃSuspected meningitis	97	49 (40–60)	18	9 (5–14)
ȃSuspected septicemia	48	24 (18–32)	1	1 (<1–2)
ȃRadiological pneumonia with consolidation	200	90 (78–104)	3	1 (<1–4)
Age 10–14 y				
ȃSuspected pneumonia	170	102 (87–119)	5	3 (1–7)
ȃSuspected meningitis	17	10 (6–16)	1	1 (<1–3)
ȃSuspected septicemia	17	10 (6–16)	0	ND
ȃRadiological pneumonia with consolidation	63	37 (28–47)	1	1 (<1–3)
Age 15–54 y				
ȃSuspected pneumonia	500	84 (77–91)	21	4 (2–5)
ȃSuspected meningitis	18	3 (2–5)	2	<1 (<1–1)
ȃSuspected septicemia	44	7 (5–10)	0	ND
ȃRadiological pneumonia with consolidation	164	26 (22–30)	13	2 (1–4)
Age ≥55 y				
ȃSuspected pneumonia	105	123 (100–149)	16	19 (10–31)
ȃSuspected meningitis	3	4 (1–10)	1	1 (<1–7)
ȃSuspected septicemia	2	2 (0–9)	0	ND
ȃRadiological pneumonia with consolidation	55	61 (46–80)	10	11 (5–20)
Culture positive	144	13 (11–15)	14	1 (<1–2)
ȃ*Streptococcus pneumoniae*	84	8 (6–10)	6	1 (<1–1)
ȃ*Neisseria meningitidis*	16	2 (1–2)	3	<1 (<1–1)
ȃ*Staphylococcus aureus*	15	1 (1–2)	2	<1 (<1–1)
ȃ*Salmonella* spp	7	1 (<1–1)	0	ND
ȃOther gram negatives	22	2 (1–3)	3	<1 (<1–1)

Abbreviations: CI, confidence interval; ND, not determined (no reported deaths in the study therefore incidence cannot be determined); PY, person-years.

Cases of suspected pneumonia, meningitis, or septicemia are counted separately while radiological pneumonia is a subset of suspected pneumonia.

All results in the table have been rounded to the nearest whole number except where the nearest whole number is zero, in which case <1 has been used.

The minimum estimated annual mortality rate associated with suspected meningitis, septicemia, or pneumonia was 7 per 100 000 PY with the greatest mortality being 20 per 100 thinsp;000 PY in those aged ≥55 years. Based on counts of all-cause mortality in the BHDSS during the study period, we estimated that suspected meningitis, septicemia, or pneumonia was associated with a minimum of 3.6% of all deaths in 5- to 9-year-olds, 1.8% in 10- to 14-year-olds, 3.5% in 15- to 54-year-olds, 1.5% in those aged ≥55 years, and 2.7% of all deaths in those aged ≥5 years.

## DISCUSSION

Eight years of population-based surveillance among older children and adults in rural Gambia revealed a significant burden of disease due to pneumonia, meningitis, and septicemia. The findings, generated using standardized procedures in a well-characterized population under demographic surveillance, provide a representative description of the frequency, etiology, and mortality associated with meningitis, septicemia, and pneumonia in age groups for which there are few available data in Africa. Please see the Supplementary Material for more detail on the context of this discussion and a comparison of publications referenced in Supplementary Material Table 1.

The minimum incidence and mortality of suspected pneumonia, meningitis, or septicemia was substantial. We found that suspected pneumonia was >5 times more prevalent than suspected meningitis or suspected septicemia and that the greatest burden of any illness occurred in the age group 5–9 years. Our observed minimum incidence of suspected pneumonia of 133 cases per 100 000 PY is a similar figure to Cohen et al [13] studying severe respiratory infection in Johannesburg, and our minimum incidence for suspected meningitis of 13 cases per 100 000 PY is similar to Traore et al [18] and Soeters et al [15] studying meningitis in West Africa. Thriemer et al in Zanzibar [17] reported a bacteremia incidence in the age groups 5–15 years and >15 years of 6 and 7 per 100 000 PY, respectively, approximately half our value of 13, whereas Sigauque et al in Mozambique [14] reported a rate of 49 cases of bacteremia per 100 000 PY in 5- to 15-year-olds, a rate not dissimilar from rates we report when the age discrepancy is taken into account. Final rates from Thriemer et al [17], adjusted for study nonenrollment and blood culture sensitivity, are 20 times higher, and from Verani et al [19] (the only similar study to adjust for healthcare-seeking behavior), 2–3 times higher than their unadjusted rates. This emphasizes the point that nearly all such estimates are of a minimum incidence. However, the fact that our surveillance operated in all primary healthcare centers in the surveillance area, the high enrollment rate in surveillance, and the use of standardized physician diagnoses rather than positive laboratory tests are likely to contribute to a robust figure that omits only a healthcare-seeking behavior adjustment.

Our data suggest that pneumonia, meningitis, and septicemia were together associated with at least 2.*7*% of all deaths in older children and adults in the study population. Case fatality risk (CFR) was highest in cases of suspected meningitis (16*.3*%), although due to its higher prevalence across all ages, suspected pneumonia accounted for double the deaths of suspected meningitis and was particularly lethal in older patients (CFR of 15.2% at 4 months). Cohen et al [13] reported an in-hospital CFR of 5% in HIV-uninfected individuals with pneumonia, compared to our risk of 2% for admitted cases of suspected pneumonia in our low-HIV-incidence cohort. Traore et al [18] reported a CFR for pneumococcal meningitis of 45% in 5- to 14-year-olds and 44% in subjects aged >14 years, whereas our mortality risk in these 2 age groups was 19% and 6%, respectively, for suspected meningitis, most of whom had no microbiological diagnosis.

Across all diseases and ages, we found a concerningly high mortality in the 4-month period after discharge from hospital, with 40 postdischarge deaths compared to 28 inpatient deaths. Postdischarge mortality was particularly associated with suspected pneumonia and with consolidation on chest radiograph: 32 of the 40 postdischarge deaths were associated with suspected pneumonia and 16 with radiological pneumonia. Postdischarge mortality was similar across all 4 age groups [27]. These observations suggest that postdischarge death may relate to inadequate diagnosis and triage and/or therapy rather than a group-specific predisposing factor. Other plausible explanations, some of which have been highlighted in younger children [27], include unaddressed comorbid conditions such as malnutrition, anemia, HIV, tuberculosis, sickle cell disease, or diabetes, or issues such as nonmedical discharge or nosocomial infection. Pneumococcal antimicrobial resistance is not significant in our setting [28].

We and Sigauque et al [14] show similar trends in the bacteria causing bacteremia in older children and adults in sub-Saharan Africa; *S. pneumoniae*, *S. aureus*, and *Salmonella* species featured prominently in each study. We have fewer gram negatives in total: 32% vs 39% of isolates, and 8% vs 20% for *Salmonella* species. This may reflect our screening criteria, which did not include screening for abdominal or diarrheal causes of sepsis or the differing burden of HIV or *Salmonella* species disease in the 2 communities. Our study and all studies reviewed show that most patients investigated with blood, CSF, and lung cultures have no microbiological diagnosis made, even in patients who died from meningitis and who are likely to have had a bacterial infection. In our study, culture-positive *S. pneumoniae* was due to serotypes 1 and 5 in 78% of cases, while 90% of cases were caused by serotypes included in 13-valent PCV, the PCV currently used in The Gambia’s infant immunization program. Although the effects of herd protection from infant pneumococcal vaccination during our study period are uncertain [21], this finding may have implications for the proportion of invasive pneumococcal disease potentially preventable by protection related to PCV.

The strengths of our study include the standardization of screening, clinical investigation, case definitions, and laboratory practices, with investigation of >97% of eligible patients. Our surveillance included outpatients and inpatients and aimed to detect all cases presenting to any government health facility. Conduct of the study in a geographically and demographically defined population allowed follow-up after discharge, calculation of incidence, and estimation of the proportion of all deaths that were related to pneumonia, meningitis, or septicemia. Limitations included the inability to detect all cases that occur in the population, particularly in those who seek care outside the government healthcare sector (eg, from a traditional healer or a pharmacy) or who die at home, and absence of healthcare-seeking behavior data to correct for these patients; less-than-optimal sensitivity of conventional microbiology to detect all bacterial pathogens; and no testing for mycobacterial, viral, or other pathogens.

Our study demonstrates the extent to which sepsis syndromes remain major causes of death and disease in older children and adults in rural Africa and the many gaps that exist in understanding the causes of these syndromes and determinants of their outcome in older children and adults. High rates of postdischarge mortality in Africa have been observed in young children [27], but to our knowledge this is the first description of postdischarge mortality associated with acute infections exceeding inpatient mortality in older children and adults. This finding underlines the importance of follow-up of pneumonia patients, apparently cured, with even closer attention paid to those with consolidation on chest radiograph. The determinants of postdischarge mortality in older patients should be formally studied in other African settings to establishing prevention strategies. The inadequacy of conventional diagnostics was brought into sharp relief, highlighting the need for better diagnostics to determine the etiology of pneumonia, meningitis, or septicemia and prevalent comorbidities. We call for a greater focus on the prevention, diagnosis, treatment, and follow-up of sepsis patients by the international community.

## Supplementary Data

Supplementary materials are available at *Clinical Infectious Diseases* online. Consisting of data provided by the authors to benefit the reader, the posted materials are not copyedited and are the sole responsibility of the authors, so questions or comments should be addressed to the corresponding author.

## Supplementary Material

ciac603_Supplementary_Data

## Data Availability

Deidentified participant data will be made available upon request and application to The Gambia Government/MRC Joint Ethics Committee (www.mrc.gm). Access criteria will include demonstrated capacity to analyze such data with reasoned interpretation.

## Notes

***Acknowledgments.*** Surveillance was conducted at the Basse Major Health Centre and government health facilities in Gambissara, Demba Kunda, Fatoto, Garawol, Sabi, and Koina. We thank all staff of the Medical Research Council (MRC) Basse Field Station and the residents of the Basse Health and Demographic Surveillance System for supporting the study.

***Author contributions.*** G. A. M., P. C. H., and B. G. designed and established the surveillance system. I. M. H., M. N., S. M. S., E. W. G., and Y. A. O. provided clinical care, investigated the patients, and supervised the demographic surveillance. H. B., A. M., and R. S. performed the microbiology. L. C. supervised the health facilities in the study area and provided Ministry of Health support. E. W. G. and G. A. M, verified the underlying data, performed the analysis, and drafted the manuscript. E. W. G., G. A. M., I. M. H., S. M. S., M. N., and Y. A. O. performed the literature search. All the authors had access to the data and accept responsibility to submit for publication. All authors reviewed and approved the final manuscript.

***Disclaimer.*** None of the funding sources were involved in data collection, analysis, or interpretation.

***Financial support.*** This work was supported by Gavi The Vaccine Alliance's Pneumococcal Vaccines Accelerated Development and Introduction Plan; the Bill & Melinda Gates Foundation (grant number OPP1020372); and the UK MRC.
