# Supplementary material for: Pneumonia, Meningitis, and Septicemia in Adults and Older Children in Rural Gambia: 8 Years of Population-Based Surveillance
Source: Clin Infect Dis. 2022 Jul 29;76(4):694–703. doi: 10.1093/cid/ciac603 (PMC9938739; doi:10.1093/cid/ciac603)
Supplement: ciac603_Supplementary_Data [file ciac603_supplementary_data.zip › 220701 PSP_A5_SBI_Epi_v14.1_appendices.docx]

**Appendix 1·1**

Referral criteria applied by nurses for patents presenting to surveillance area health care facilities

| **Criteria C** |
| --- |
| **Patients aged 5 years and over are to be referred for assessment if one or more of the following have been present for 14 days or less (unless acute pneumonia, meningitis or septicaemia is otherwise suspected):** |
| 1. **History of cough and difficulty breathing**      1. **History of cough and pleuritic chest pain**      1. **History of cough and supraclavicular/sternal recession or nasal flaring**      1. **History of productive cough and fever**      1. **History of rigors**      1. **History of seizure**      1. **Impaired consciousness**      1. **Altered mental state**      1. **Axillary temperature of at least 38°C, or less than 36˚C, in a patient admitted or being admitted.**      1. **Photophobia**      1. **Neck stiffness**      1. **Local musculoskeletal swelling or tenderness**      1. **Irrespective of residential location, any patient with**   **suspected meningitis** |

**Cough** includes a dry cough, sputum production, or haemoptysis.

**Impaired consciousness**: V, P, or U on the AVPU score, where A is if the patient is alert, V if responsive to verbal stimulus, P if responsive to pain stimulus, and U if unresponsive.

If patients require antibiotics before referral, a blood culture will be collected before antibiotic administration. Patients being referred will have malaria tests in Basse. Patients being admitted from August until December should have a rapid diagnostic test for malaria.

**Appendix 1·2**

Clinical criteria used by study doctors for diagnosing suspected pneumonia, meningitis or septicaemia

| **Criteria G** |
| --- |
| **Clinical criteria for the diagnosis of suspected meningitis in patients aged 5 years and over:** |
| **Suspected meningitis will be defined as a physician diagnosis, and is also to be considered if two or more of the following are present:**     1. **Axillary temperature ≥37.5°C**      1. **Meningism (neck stiffness and/or photophobia)**      1. **Altered mental state (Glasgow coma scale <14)** |

| **Criteria H** |
| --- |
| **Clinical criteria for the diagnosis of suspected pneumonia in patients aged 5 years and over:** |
| **Suspected pneumonia will be defined as a physician diagnosis, and according to clinical judgement, as an illness of 14 days’ duration or less where 2 or more of the following are present:**     1. **Cough**      1. **Haemoptysis**     **4. Pleuritic chest pain**     1. **Breathlessness**      1. **Axillary temperature of 38.0˚C or greater** |

**Those with recurrent wheeze** may be investigated according to clinical judgement.

| **Criteria I** |
| --- |
| **Clinical criteria for the diagnosis of suspected septicaemia in patients aged 5 years and over:** |
| **Suspected septicaemia will be defined as a physician diagnosis or at least one of:**     1. **Clinician diagnosis of focal sepsis (including but not limited to:**   **septic arthritis, osteomyelitis, endocarditis, peritonitis, liver abscess, soft tissue abscess or cellulitis), or of generalised septicaemia**     1. **Axillary temperature <36.0˚C or ≥38˚C in a patient admitted or being admitted**      1. **History of rigors** |

**Appendix 1·3**

Standardised investigation according to suspected disease

1. All patients are to have blood culture.
2. All patients are to have,
   1. a rapid diagnostic test for malaria (from January – July the RDT is only done if the surveillance number ends in ‘0’).
   2. serum collection for antibiotic activity detection if the surveillance number ends in ‘0’ or ‘5’ and the patient is enrolled in Basse.
3. Patients with suspected meningitis are to have lumbar puncture.
4. Patients with suspected pneumonia are to have chest X-ray.
5. Chest X-ray should also be considered in patients with meningitis or septicaemia if the clinician’s impression is of co-existing pneumonia or if it is judged that a chest X-ray will assist in management.
6. Lung aspirate should be considered for a patient if peripheral consolidation has been demonstrated, preferably by X-ray.
7. Other investigations including pleural tap and joint aspirate may be considered according to the clinical indication.
