# Supplementary material for: Pneumonia, Meningitis, and Septicemia in Adults and Older Children in Rural Gambia: 8 Years of Population-Based Surveillance
Source: Clin Infect Dis. 2022 Jul 29;76(4):694–703. doi: 10.1093/cid/ciac603 (PMC9938739; doi:10.1093/cid/ciac603)
Supplement: ciac603_Supplementary_Data [file ciac603_supplementary_data.zip › PSP_A5_SBI_Epi_literature review supplement_V14.1.docx]

**Literature review supplementary material**

**Background**

To contextualise our findings, we performed a literature review for similar studies. Here we present the results of our search and our synthesis of the evidence.

**Method**

We searched pubmed, embase and Web of Science for the following terms and using the following filters and search terms:

Classical Article, Clinical Study, Clinical Trial, Controlled Clinical Trial, Journal Article, Meta-Analysis, Multicenter Study, Observational Study, Pragmatic Clinical Trial, Randomized Controlled Trial, Review, Systematic Reviews, Publication date from 2016/04/02 to 2019/02/30, Humans, Child: 6-12 years, Adolescent: 13-18 years, Adult: 19+ years, Young Adult: 19-24 years, Adult: 19-44 years, Middle Aged + Aged: 45+ years, Middle Aged: 45-64 years, Aged: 65+ years, 80 and over: 80+ years.

 Search terms:

(meningitis OR sepsis OR septic* OR bacteremia OR bacteraemia OR pneumonia) AND (south africa OR gambia OR kenya OR namibia OR senegal OR guinea OR sierra leone OR ivory OR d'ivoire OR benin OR togo OR burkina faso OR mali OR chad OR niger OR cameroon OR nigeria OR ghana OR gabon OR congo OR central african OR rwanda OR burundi OR uganda OR tanzania OR malawi OR mozambique OR angola OR zambia OR botswana OR lesotho OR zimbabwe OR somalia OR ethiopia OR sudan OR mauritania OR eritrea OR djibouti OR liberia) AND ((risk*[Title/Abstract] OR risk*[MeSH:noexp] OR risk *[MeSH:noexp] OR cohort studies[MeSH Terms] OR group[Text Word] OR groups[Text Word] OR grouped [Text Word]) OR (incidence[MeSH:noexp] OR mortality[MeSH Terms] OR follow up studies[MeSH:noexp] OR prognos*[Text Word] OR predict*[Text Word] OR course*[Text Word]))

Co-authors then screened titles and abstracts for articles that were original research or review articles and:

- Were based in Africa,
- Were prospective
- Did not recruit solely HIV positive patients
- Collected data for at least one year
- Contained population denominators to calculate adjusted or unadjusted disease or syndrome incidence
- Collected specimens from sterile sites
- Were investigating pneumonia and, or meningitis and, or sepsis
- Were investigating people aged five years and older or had data from these age groups which could be disaggregated from data of those under five
- Were performed after 2000

Each article list was reviewed by a single co-author who, after reviewing the full text made the final inclusion and exclusion decision.

**Results**

**Description of studies found:**

We found eight studies, which included data from 11 countries, between them representing Western (Mali, Togo, Niger, Burkina Faso)(1-3), Central (Chad)(1),Eastern (Kenya and Zanzibar(4-6)) and southern Africa (Malawi, Mozambique(7, 8)). Sites were a mixture of rural and urban. HIV prevalence ranged from unknown, to low (4%), to high (74%). Two studies included children up to 15 years but no adults(2, 8), four studies included children over four and adults(1, 3, 5, 6) and one study included only participants over 14(7). Study duration was between one and five years. Two of the eight studies focussed on invasive bacterial infections regardless of cause or site(5, 8), six, focussed either on a single syndrome (severe acute respiratory syndrome, meningitis)(1, 3, 4) or a single organism or genus (*Streptococcus pneumoniae, Haemophilus influenzae* type b, non-typhoidal salmonella)(2, 3, 6, 7). However, Traore(3) and Bar-Zeev(7) published non pneumococcal aetiologies in their findings. All studies took invasive samples, for culture except one which used a molecular method for testing blood for pneumococci(4). Methods of calculating incidence varied from unadjusted incidence calculated by dividing the number of cases in each year by the catchment population of the facility(8) to adjusting for non-enrolment and non-investigated patients using fractions of positive cases in those enrolled and investigated and applying these fractions to clinically suspect cases(1). Only one study included adjustment for multiple factors which included health seeking behaviour(6). Three studies reported mortality data which could be disaggregated for the over fives(3, 4, 6). Of the three, two studies were inpatient mortality and one 30-day mortality.

**Study findings:**

Of the two studies investigating invasive bacterial infections: Sigauque et al in rural Mozambique were admitting children who had blood cultures taken if febrile or had one or more signs of severe illness. Thus, 84% of all children had a blood culture. Of children aged 5-15 years 108/1523(7%) grew a pathogen with *S. pneumoniae* constituting 55% of pathogens isolated, non-typhoidal salmonellae 17%, *Staphylococcus aureus* 8%, *Haemophillus influenzae* 5%, and *Neiseria meningitides* 2%; Thriemer et al who were screening febrile patients attending four hospitals on Pemba Island, Zanzibar tested 1572 patients five years and over. 50/1572 (3%) grew a pathogen with *S*.typhi making up 72% of isolates, *S. pneumoniae* 6%, *S. aureus* 6%, *E. coli* 4% and *H. influenzae* type b 2%.

Cohen et al, taking sputum samples from those five years and older in Kalifi, Kenya for respiratory virus PCR testing and blood for PCR testing for the lytA gene of *S. pneumoniae* found 600/6519 (9%) of blood specimens positive for pneumococcus.

Soeters et al, investigating causes of meningitis by taking CSF for bacterial culture in several West African countries tested 92% of eligible patients, of whom 10853 were over five years old. They found 26% of their CSF’s returned a positive growth. They found *Neiseria meningitidis* to be the commonest pathogen followed by *S. pneumoniae* and *H. influenzae.*

Bar-Zeev et al, searching for invasive pneumococcal disease in Blantyre, Malawi in patients over 15 by taking blood and CSF cultures tested 8891 patients of whom 1724 (19.4%) were positive for a pathogen. Non-typhoidal Salmonellae constituted 36.3% of pathogens followed by *S. pneumoniae* (32.4%) *E. coli* (10.3%) and *Cryptococcus neoformans* (9.4%).

Traore et al, researching pneumococcal meningitis published non pneumococcal aetiologies of meningitis and found 571 to be positive in the over four age groups (1097/2689 specimens were positive over all age groups including those <5). Of pathogens reported S. pneumoniae comprised 50%, N. meningitidis 43% and HiB 7%.

Incidence rates of bacterial infections, and the methods to calculate them vary widely and are presented in table 1. For syndrome-based surveillance SARI admissions in a cohort with high rates of HIV positivity in Kalifi, Kenya were up to 617/100,000/year and for meningitis in the meningitis belt of West Africa up to 18/100.000/year. Rates of blood stream infection varied between 325/100,000/year for invasive non-typhoidal salmonella disease in rural Kenya to rates of 1.5/100,000/year for invasive HiB disease in urban Bamako. What is clear is that most of the rates reported are a minimum value and that true rates of disease are likely to be substantially higher.

Death rates for adults and children over four years vary between 45% for children with pneumococcal meningitis to 7% for those with severe acute respiratory illness – albeit in a cohort with a high HIV prevalence.

**Conclusion and discussion**

Severe bacterial infections including pneumonia, meningitis, and septicaemia cause significant morbidity and mortality in older children and adults in West Africa. Many of the agents causing these deaths are well established but prevention is hampered by the number of aetiological agents and their ubiquity in the environment. Our study shows a substantial burden of invasive bacterial diseases in older children and adults in West Africa over a long period of surveillance with unique estimates of minimum incidence derived from prospective surveillance and population data. For the first time, we show substantial post-discharge mortality in this patient group. We add new findings on the fractions of bacterial pathogens causing the syndromes of pneumonia, meningitis, and septicaemia. These data will inform global burden of disease estimates, highlight disease aetiology and therefore inform management and draw attention to the need for careful risk assessment and follow up of patients. Research is needed to improve diagnostics and to establish non-bacterial aetiologies of severe infections in older children and adults in low-income countries, to address underlying poor prognostic factors and establish evidence-based follow-up strategies to address post discharge mortality.

**References:**

1. Soeters HM, Diallo AO, Bicaba BW, Kadade G, Dembele AY, Acyl MA, et al. Bacterial Meningitis Epidemiology in Five Countries in the Meningitis Belt of Sub-Saharan Africa, 2015-2017. J Infect Dis. 2019;220(220 Suppl 4):S165-S74.

2. Sow SO, Diallo S, Campbell JD, Tapia MD, Keita T, Keita MM, et al. Burden of invasive disease caused by Haemophilus influenzae type b in Bamako, Mali: impetus for routine infant immunization with conjugate vaccine. The Pediatric infectious disease journal. 2005;24(6):533-7.

3. Traore Y, Tameklo TA, Njanpop-Lafourcade BM, Lourd M, Yaro S, Niamba D, et al. Incidence, seasonality, age distribution, and mortality of pneumococcal meningitis in Burkina Faso and Togo. Clin Infect Dis. 2009;48 Suppl 2:S181-9.

4. Cohen C, Walaza S, Moyes J, Groome M, Tempia S, Pretorius M, et al. Epidemiology of severe acute respiratory illness (SARI) among adults and children aged >/=5 years in a high HIV-prevalence setting, 2009-2012. PLoS One. 2015;10(2):e0117716.

5. Thriemer K, Ley B, Ame S, von Seidlein L, Pak GD, Chang NY, et al. The burden of invasive bacterial infections in Pemba, Zanzibar. PLoS One. 2012;7(2):e30350.

6. Verani JR, Toroitich S, Auko J, Kiplang'at S, Cosmas L, Audi A, et al. Burden of Invasive Nontyphoidal Salmonella Disease in a Rural and Urban Site in Kenya, 2009-2014. Clin Infect Dis. 2015;61 Suppl 4:S302-9.

7. Bar-Zeev N, Mtunthama N, Gordon SB, Mwafulirwa G, French N. Minimum incidence of adult invasive pneumococcal disease in Blantyre, Malawi an urban african setting: a hospital based prospective cohort study. PLoS One. 2015;10(6):e0128738.

8. Sigauque B, Roca A, Mandomando I, Morais L, Quinto L, Sacarlal J, et al. Community-acquired bacteremia among children admitted to a rural hospital in Mozambique. The Pediatric infectious disease journal. 2009;28(2):108-13.
