# Supplementary material for: Pneumonia, Meningitis, and Septicemia in Adults and Older Children in Rural Gambia: 8 Years of Population-Based Surveillance
Source: Clin Infect Dis. 2022 Jul 29;76(4):694–703. doi: 10.1093/cid/ciac603 (PMC9938739; doi:10.1093/cid/ciac603)
Supplement: ciac603_Supplementary_Data [file ciac603_supplementary_data.zip › PSP_A5_SBI_Epi_literature review supplement_V14.1__table 1.docx]

**Table 1. Incidence rates and mortality rates of syndrome or bacterial infection in selected studies**

| Study | Setting | Organism or syndrome | Incidence per 100,000 population per year | How calculated or adjusted | Mortality rate, if available |
| --- | --- | --- | --- | --- | --- |
| Cohen et al 2015 | Urban Johannesburg, South Africa, (74% HIV incidence) | SARI* hospital admissions | 325 (in 2012) (99 in HIV uninfected) to 617 (in 2010) (194 in HIV uninfected) | Adjusted for non-enrolment and non-attendance but not for HSB^†^ | 7% in hospital mortality |
| Verani et al 2015 | Rural and urban Kenya | Invasive non-typhoidal salmonella disease | 62.2 in 18-49 year olds in urban setting  325 in 18-49 year olds in rural setting | Adjusted for non-enrolment, and HSB | At 30 days: 1.4% for urban, 2.9% rural |
| Thriemer et al 2012 | Rural and urban Pemba island, Zanzibar | Bacterial blood stream infections | 162 in 5-14 year old  171 in >14 year old | Adjusted for study non enrolment and blood culture sensitivity |  |
| Bar-Zeev et al 2015 | Urban Blantyre, Malawi | Invasive pneumococcal disease with positive blood culture or CSF for pneumococci | 60.6 in those over 14 | Unadjusted |  |
| Sigauque et al 2009 | Rural Mozambique | Bacterial blood stream infections | 49 | Unadjusted |  |
| Soeters et al 2019 | Rural and urban Chad, Mali, Burkina Faso, Niger, and Togo | Bacterial meningitis | Incidence varied between 5-18 in age groups >4 | Adjusted for non-enrolment and for non-investigation |  |
| Traore et al 2009 | Rural and urban Burkina Faso and Togo | Pneumococcal meningitis | 10 in 5-14 years old and 11 in >14 years old | Unadjusted | 45% in 5 to <15 year olds 44% in >14 year olds |
| Sow et al 2005 | Urban Bamako, Mali | Invasive *H. influenzae* type B disease | 1.5 | Adjusted using rates of febrile admissions and rate of HiB disease in the febrile study population |  |

* SARI: severe acute respiratory infection

^†^ HSB: health seeking behaviour
